# Interleukin-17 promotes nitric oxide-dependent expression of PD-L1 in mesenchymal stem cells

**DOI:** 10.1186/s13578-020-00431-1

**Published:** 2020-05-26

**Authors:** Shijia Wang, Guan Wang, Liying Zhang, Fengying Li, Keli Liu, Ying Wang, Yufang Shi, Kai Cao

**Affiliations:** 1 Key Laboratory of Stem Cell Biology, Shanghai Jiao Tong University School of Medicine (SJTUSM) & Shanghai Institutes for Biological Sciences (SIBS), Academy of Sciences (CAS), Shanghai, 200031 China; 2 CAS Key Laboratory of Tissue Microenvironment and Tumor, Shanghai Institute of Nutrition and Health, Shanghai Institutes for Biological Sciences, University of Chinese Academy of Sciences, Chinese Academy of Sciences, Shanghai, 200031 China; 3 The First Affiliated Hospital of Soochow University, State Key Laboratory of Radiation Medicine and Protection, Institutes for Translational Medicine, Soochow University, Suzhou, 215123 China

**Keywords:** Mesenchymal stem/stromal cells, Programmed death-ligand 1, Interleukin-17, Nitric oxide, Tumor microenvironment

## Abstract

**Background:**

Interleukin-17A (IL-17) is an evolutionary conserved cytokine and best known for its role in boosting immune response. However, recent clinical researches showed that abundant IL-17 in tumor microenvironment was often associated with poor prognosis and reduced cytotoxic T cell infiltration. These contradictory phenomena suggest that IL-17 may have unique target cells in tumor microenvironment which switch its biological consequences from pro-inflammatory to anti-inflammatory. Mesenchymal stem/stromal cells (MSCs) are a major component of the tumor microenvironment. Upon cytokine stimulation, MSCs can express a plenary of inhibitory molecules, playing a critical role in tumor development and progression. Therefore, we aim to investigate the role of IL-17 in MSC-mediated immunosuppression.

**Results:**

We found IFNγ and TNFα, two major cytokines in tumor microenvironment, could induce programmed death-ligand 1 (PD-L1) expression in MSCs. Interestingly, IL-17 has a synergistic effect with IFNγ and TNFα in elevating PD-L1 expression in MSCs. The presence of IL-17 empowered MSCs with strong immunosuppression abilities and enabled MSCs to promote tumor progression in a PD-L1 dependent manner. The upregulated PD-L1 expression in MSCs was due to the accumulation of nitric oxide (NO). On one hand, NO donor could mimic the effects of IL-17 on MSCs; on the other hand, IL-17 failed to enhance PD-L1 expression in inducible nitric oxide synthase (iNOS) deficient MSCs or with iNOS inhibitor presence.

**Conclusions:**

Our study demonstrates that IL-17 can significantly increase the expression of PD-L1 by MSCs through iNOS induction. This IL-17-MSCs-PD-L1 axis shapes the immunosuppressive tumor microenvironment and facilitates tumor progression.

## Background

Interleukin-17A (IL-17) is the first member discovered in the IL-17 cytokine family [1]. It is primarily produced by Th17 cells during immune responses and exerts strong pro-inflammatory functions in infectious, inflammation and autoimmune diseases [2, 3]. The great success of tumor immunotherapies highlights the roles of immune responses in tumor progression, in which the role of IL-17 has also drawn much attention [4, 5]. Th17 cells have been found in various types of human cancers and different experimental animal tumor models [6–8]. Despite its superior abilities in augmenting immune response, both clinical studies and animal models suggests Th17-related cytokines, such as IL-17 and IL-23, can promote tumor progression [9, 10]. Currently, the tumor promoting effects of IL-17 are regarded as its effects on tumor cells and vascular endothelial cells [11, 12]. However, the role of other components of tumor stroma in IL-17-mediated modulation of tumor growth remains obscure.

In solid tumors, tumor stroma provides fertile soil for tumor growth and thus plays important role in facilitating tumor development [13]. Mesenchymal stem cells (MSCs) are one of the major components of tumor stroma [14, 15]. MSCs are highly plastic cells and often converted to tumor associated-MSCs in tumor microenvironment, and thus obtain the abilities to promote tumor progression [14, 16]. Besides directly promoting tumor growth and angiogenesis through their secretome [14, 17], MSCs could indirectly facilitate tumor development through shaping an immunosuppressive microenvironment to help tumor escape the surveillance of immune system [18]. Upon the stimulation of pro-inflammatory cytokines, MSCs can potently suppress lymphocytes proliferation through the concerted action of chemokines and inhibitory molecules [19, 20]. Our previous works showed that tumor-associate MSCs dramatically enhanced tumor growth by recruiting suppressive immune cells to the tumor microenvironment [15, 21, 22]. MSCs could even reprogram the responsiveness of macrophages and conferred their anti-inflammatory abilities [23]. Importantly, the effects of MSCs on immune system are dependent on the cytokine milieu [18]. It is worth noting that MSCs express IL-17 receptor [24]. Therefore, IL-17 could have a role in regulating tumor associated MSCs.

Programmed death-ligand 1 (PD-L1), also known as CD274, is a B7 family protein mainly expressed on antigen presenting cells, such as macrophages and monocytes [25, 26]. PD-L1 ligation with programmed death 1 (PD-1) on activated T cells and B cells are regard as negative feedback to fine tune inflammation [27]. Dysregulated PD-L1-PD-1 pathway often leads to various inflammatory diseases, including GvHD, hepatitis, and more importantly, cancer [28, 29]. Tumor cells are found to escape immune surveillance through upregulating PD-L1 expression on their surface [30, 31]. Epidemiological investigations have proved the correlation between PD-L1 expression and tumor prognosis and great endeavor are spent to decipher the molecular mechanism of PD-L1-mediate immune suppressive pathway [25, 32]. The breakthrough in PD-L1 biology brought PD-L1-based tumor therapy to the spotlight and PD-L1 neutralizing antibodies have been approved by Food and Drug Administration for the treatment of various cancers [32]. Besides its expression on tumor cells, PD-L1 is also expressed by MSCs to exert their immunosuppressive properties [33–35]. Notably, positive correlation between IL-17 and PD-L1-expressing tumor stromal cells has been demonstrated [36]. Based on this information, it is possible that IL-17 can enhance PD-L1 expression on MSCs to promote tumor growth.

In this study, we found that IL-17 combined with IFNγ and TNFα dramatically enhanced PD-L1 expression of MSCs, compared to IFNγ and TNFα stimulation. Interestingly, we found this synergistic induction of PD-L1 was dependent on nitric oxide (NO) production by MSCs, since genetic deletion of inducible nitric oxide synthase (iNOS) and iNOS chemical inhibitor abolished this effect. Furthermore, NO donor could act as the substitute of IL-17 to promote PD-L1 expression through STAT3 pathway. To verify the biological consequence of this effect in vivo, we injected IFNγ and TNFα primed MSCs with or without IL-17 co-stimulation together with B16 melanoma to mice. We found that IL-17 significantly enhanced the tumor promoting effects of MSCs. Furthermore, this effect can be abrogated by the administration of PD-L1 neutralizing antibody, confirming the critical role of PD-L1 in this process. Our findings reveal a novel mechanism underlying the tumor promotive property of IL-17, which is through inducing PD-L1 expression on MSCs.

## Results

### IL-17 enhanced the PD-L1 expression on MSCs

**Fig. 1:**
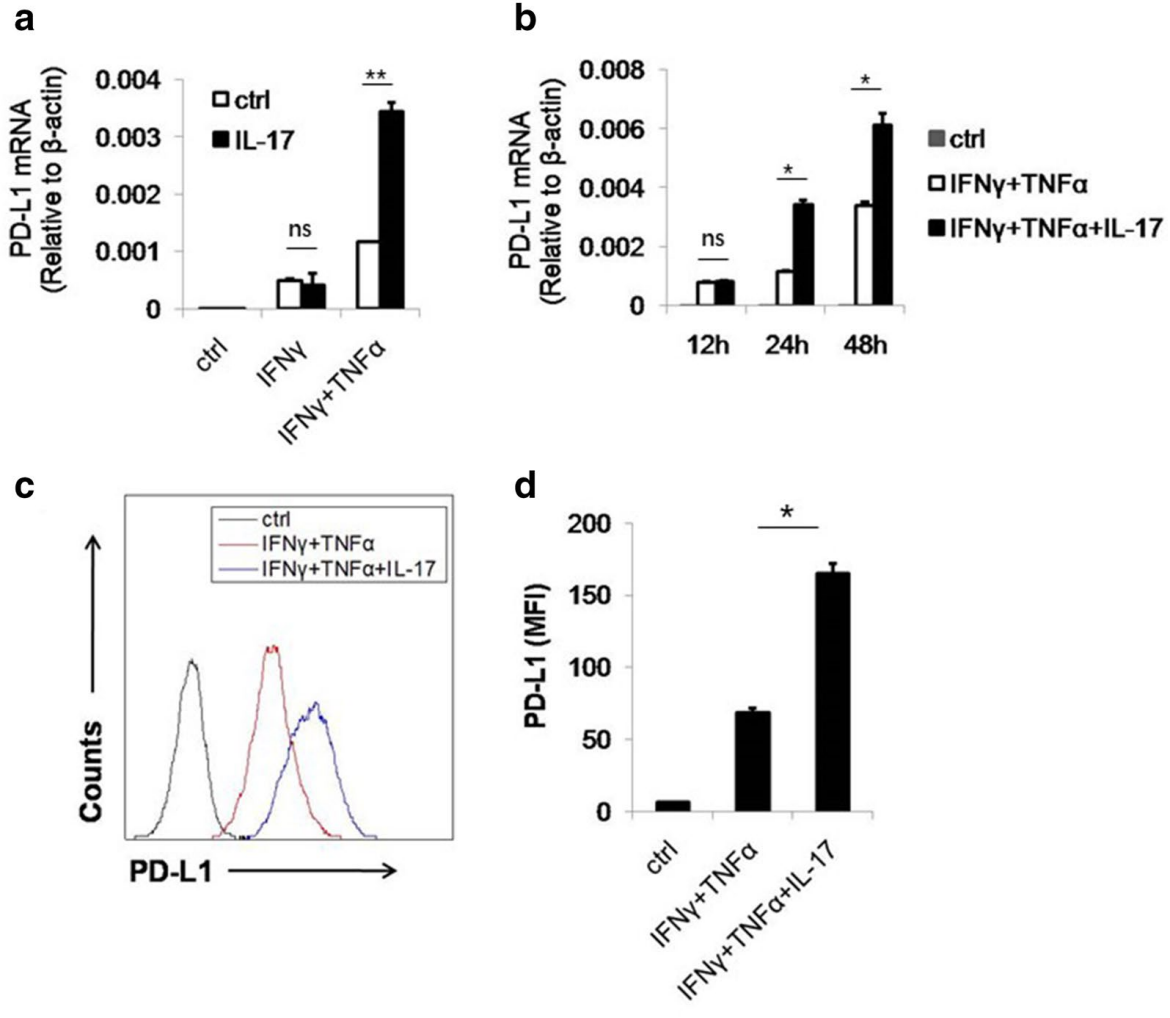
IL-17 synergizes with IFNγ and TNFα to promote PD-L1 expression on MSCs. **a** C57BL/6 MSCs were stimulated by indicated combination of cytokines. The concentration of three cytokines was 10 ng/ml each, for this and all other figures. After 24 h, cells were harvested and prepared for quantitation of PD-L1 by real-time PCR. **b** C57BL/6 MSCs were treated with different combination of cytokines. Cells were collected at the indicated time points for assay of FPD-L1 mRNA by real-time PCR. **c** C57BL/6 MSCs were treated as in **b**. After 48 h, cells were harvested and the expression of PD-L1 was determined by flow cytometry. **d** The statistical results of three separate experiments described in **c**

PD-L1 is one of the most potent immunosuppressive molecules and plays an important role in tumor development. Clinical applications of PD-L1 neutralizing antibodies have achieved great success in various cancers [37]. MSCs, a major component of the tumor microenvironment, are reported to express PD-L1 upon cytokine stimulation [34, 38]. Additionally, in the peritumoral stroma of hepatocellular carcinoma (HCC) patients, there is a positive correlation between IL-17 producing cells and PD-L1 expressing cells, indicating a possible relationship between IL-17 and stromal PD-L1 expression [36]. To test this hypothesis, we stimulated MSCs with IL-17 in vitro. However, IL-17 could not directly induce PD-L1 expression on MSCs (Fig. 1a). Since another important function of IL-17 is to synergize with other cytokines to upregulate gene expression [12, 39]. We tested whether IL-17 could further enhance the PD-L1 expression induced by IFNγ and TNFα [34, 40]. We found that in this scenario, IL-17 dramatically enhanced PD-L1 expression (Fig. 1a). Furthermore, the effect of IL-17 was not observed at 12 h, suggesting that the PD-L1 upregulation might be a secondary effect of IL-17 (Fig. 1b). To confirm this synergized effect of IL-17, we next examined the expression of PD-L1 on protein level. As expect, IL-17 combined with IFNγ and TNFα also elevated PD-L1 protein level on the surface of MSCs (Fig. 1c, d). Taken together, IL-17 and inflammatory cytokines, acting in concert, can promote the expression of PD-L1 on MSCs.

### Nitric oxide is indispensable for the synergistic effect of IL-17 on PD-L1 expression

**Fig. 2:**
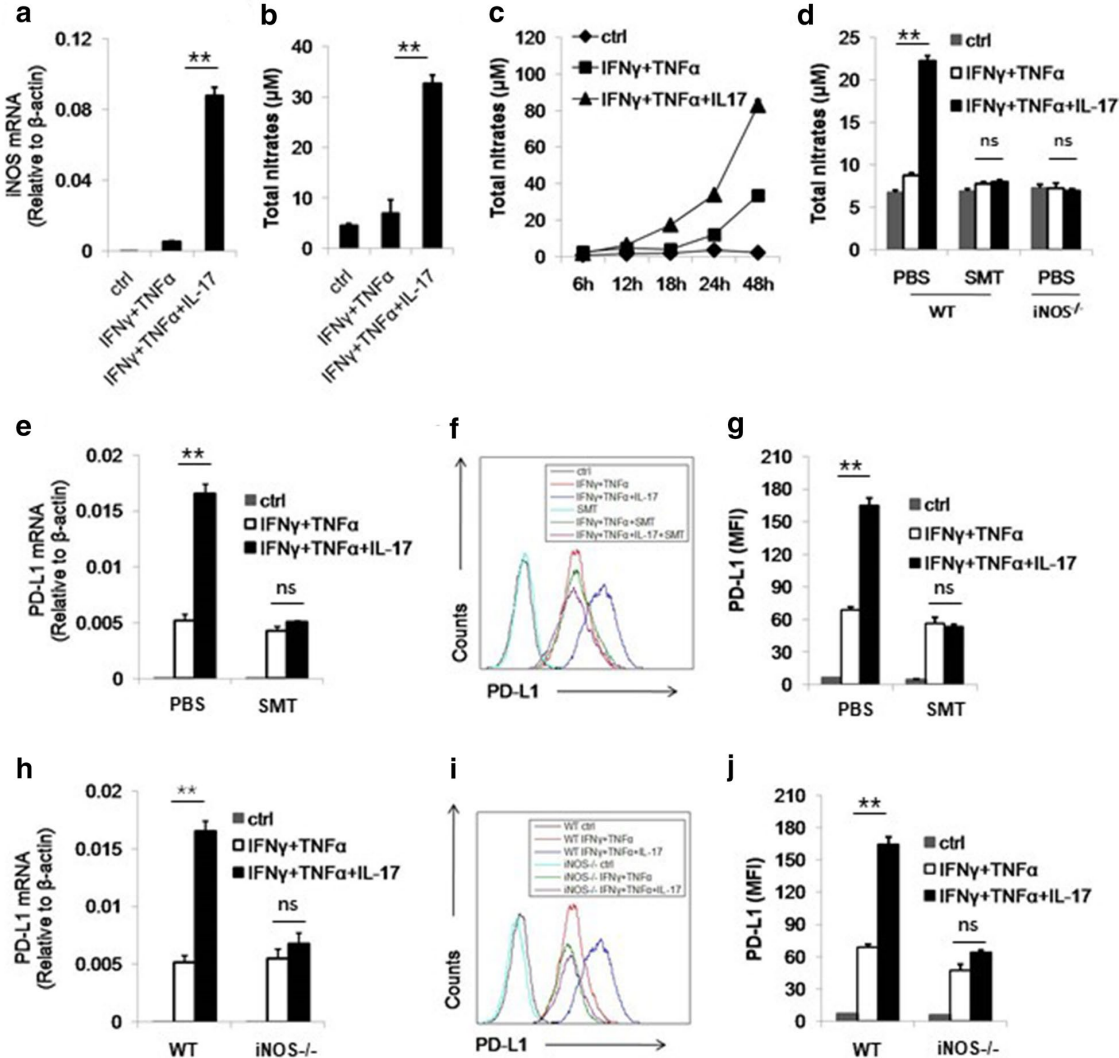
IL-17 enhances the expression of PD-L1 through NO. **a** C57BL/6 MSCs were treated by indicated cytokines for 24 h and then collected for the measurement of NO mRNA by real-time PCR. **b**, **c** C57BL/6 MSCs were treated as in A and after 24 h (**b**) or indicated time points (**c**), supernatant were collected for the assay of NO concentration by Griess reagent. **d** WT MSCs (C57BL/6 MSCs) and iNOS^−/−^ MSCs were treated by cytokines and SMT (20 mM) for 24 h and supernatant were collected for the measurement of NO. **e**, **f** C57BL/6 MSCs were treated by cytokines with or without SMT. After 24 h, cells were collected for the assay of PD-L1 mRNA by real-time PCR. After 48 h, cells were collected for the assay of PD-L1 protein by flow cytometry. **g** The statistical results of three separate experiments described in **f** are also showed. **h**, **i** WT MSCs and iNOS^−/−^ MSCs were treated with cytokines. Cells were collected for the measurement of PD-L1 mRNA and protein after 24 h and 48 h, respectively. **j** The statistical results of three separate experiments described in **i** are also showed

As we demonstrated, IL-17 did not show synergistic effect on PD-L1 expression before 12 h. Therefore, we wonder if IL-17 may act through other molecules to induce PD-L1 expression. Our previous studies have shown that IL-17 can similarly synergize with IFNγ and TNFα to induce the expression of iNOS and the production of NO [24], which is confirmed in our experiment (Fig. 2a, b). In addition, we found that the production of NO by MSCs is not detectable until 12 h (Fig. 2c). Therefore, we considered NO as the candidate of the mediator between IL-17 and enhanced PD-L1 expression. To identify the role of NO in this process, we employed iNOS inhibitor S-methylisothiourea (SMT) and iNOS knockout MSCs. It is observed that NO production by wild type (WT) MSCs was completely abolished by SMT treatment and NO was barely detectable in the supernatant of iNOS^−/−^ MSCs (Fig. 2d).

**Fig. 3:**
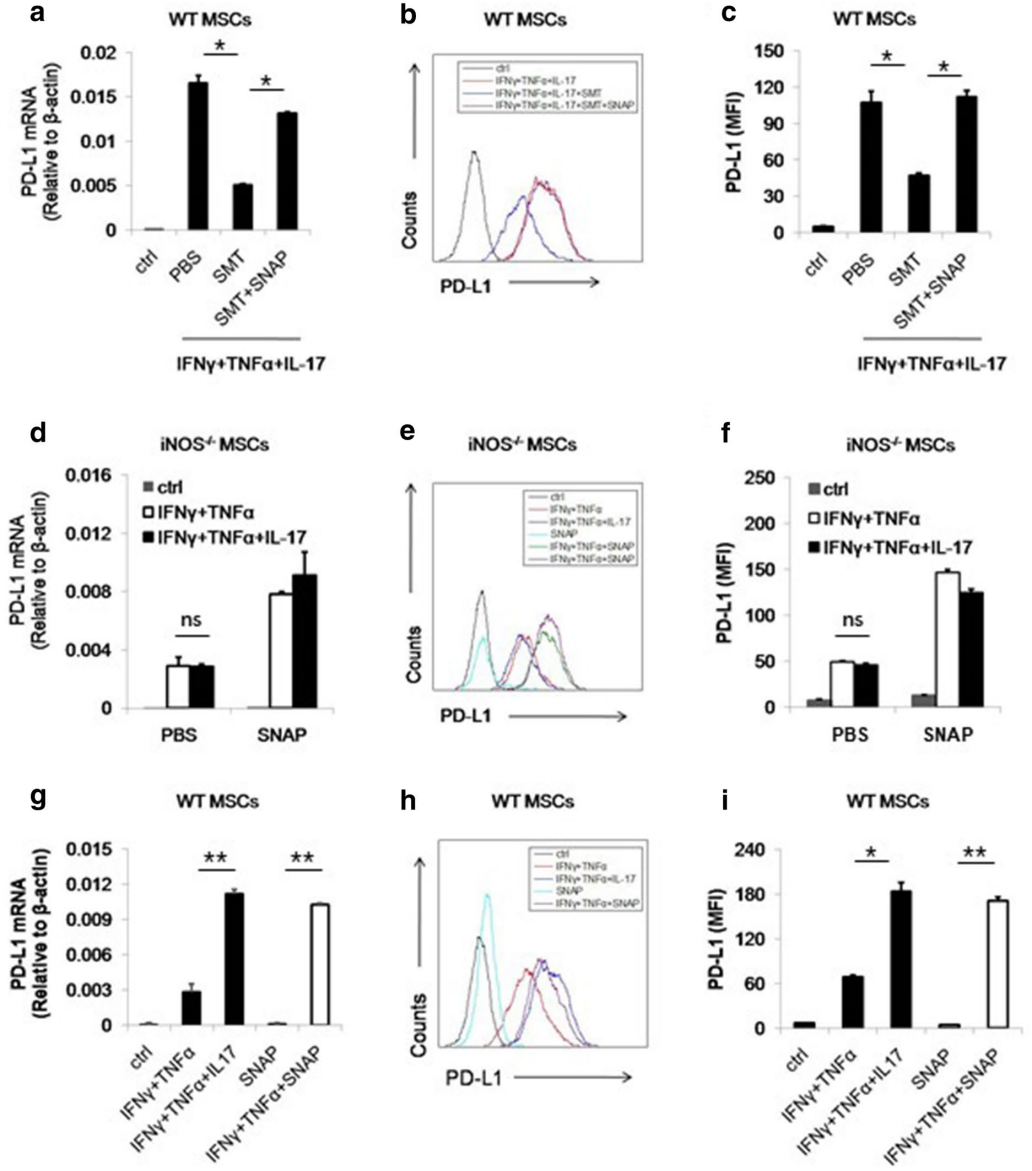
NO donor can reinstall the expression of PD-L1 on MSCs. **a**, **b** WT MSCs were treated by IFNγ, TNFα and IL-17 with or without SMT and SNAP (1 mM). The expression of PD-L1 mRNA and protein was then measured after 24 h and 48 h, respectively. **c** The statistical results of three separate experiments described in B are also showed. **d**, **e** iNOS^−/−^ MSCs were stimulated by cytokines in the presence or absence of SNAP. The expression of PD-L1 mRNA and protein was then measured after 24 h and 48 h, respectively. **f** The statistical results of three separate experiments described in **e** are also showed. **g**, **h** WT MSCs were treated with indicated combinations of cytokines. After 24 h and 48 h, the expression of PD-L1 mRNA and protein was then measured, respectively. **i** The statistical results of three separate experiments described in **h** are also showed

Furthermore, both mRNA and protein level of PD-L1 showed that the synergistic effect of IL-17 was abolished by SMT or iNOS deficiency, suggesting NO is indispensable for IL-17 to promote PD-L1 expression (Fig. 2e–g). In iNOS^−/−^ MSCs, the induction of PD-L1 by IL-17 was also abrogated, confirming the role of NO in IL-17 mediated PD-L1 enhancement (Fig. 2h–j). To further validate this point, we applied *S*-Nitroso-*N*-acetyl-DL-penicillamine (SNAP) as NO donor into MSCs culture medium. We found the inhibitory effect of SMT on PD-L1 induction was reversed on both mRNA and protein level (Fig. 3a–c). Moreover, SNAP has the similar effects on iNOS^−/−^ MSCs (Fig. 3d–f). Taken together, we demonstrated that NO mediated the promotive effect of IL-17 on PD-L1 expression of MSCs. These results also raised the possibility that NO could be the substitute of IL-17 to promote PD-L1 expression on MSCs. To test this hypothesis, we added SNAP with IFNγ and TNFα in culture medium of MSCs. Interestingly, we found that the combination of these three molecules can stimulate the expression of PD-L1 to the same level of IFNγ, TNFα and IL-17 treated MSCs (Fig. 3g–i). Notably, SNAP alone could not enhance the expression of PD-L1, indicating certain signaling pathways activated by IFNγ and TNFα were required for NO to exert its function on MSCs. Thus, we concluded that IL-17-induced NO acts in concert with IFNγ and TNFα signal to promote the expression of PD-L1 on MSCs.

### The effect of IL-17 on PD-L1 expression is dependent of NO-activated STAT3 phosphorylation

**Fig. 4:**
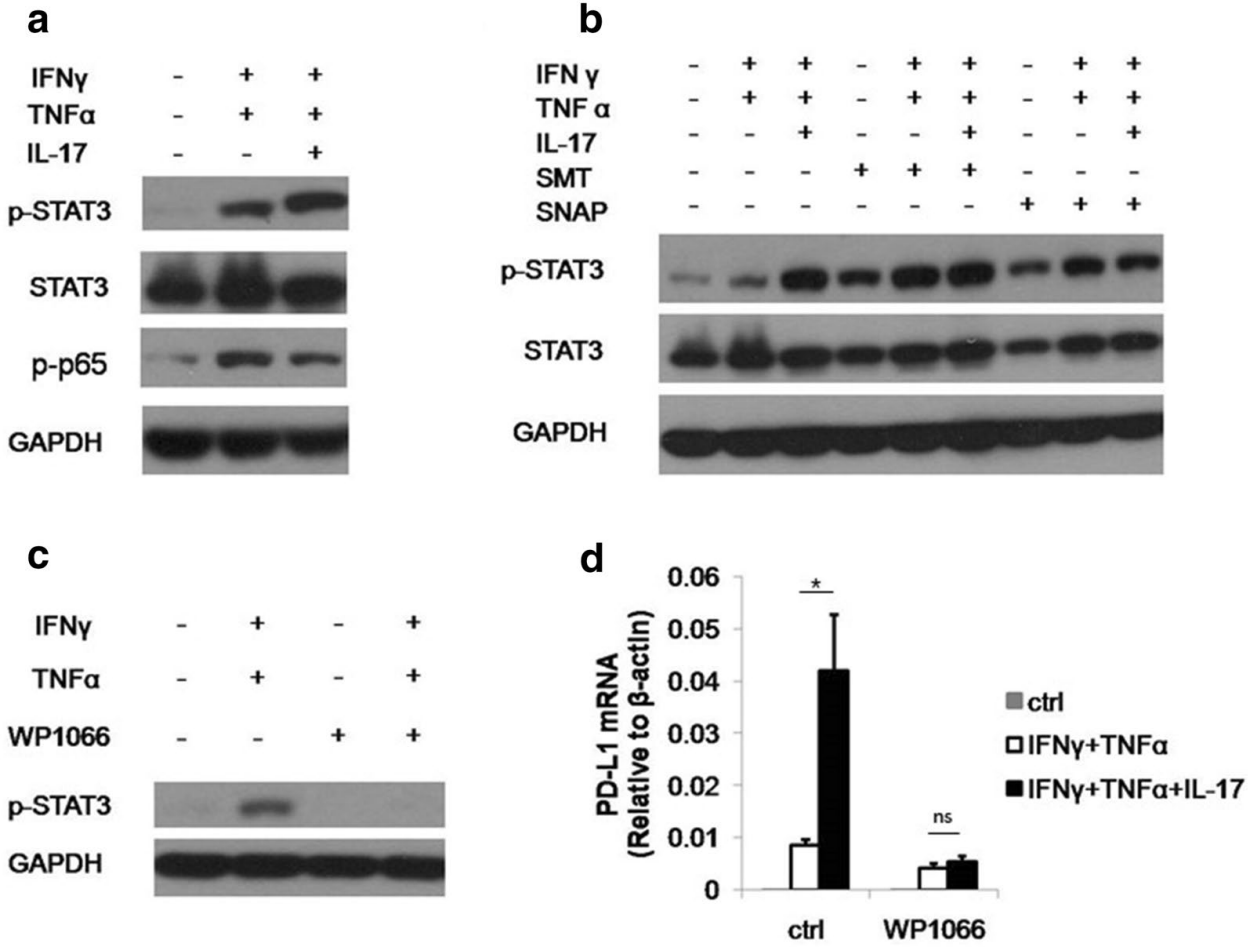
The effect of IL-17 on PD-L1 expression is dependent on NO-activated STAT3 phosphorylation. **a** C57BL/6 MSCs were treated with indicated cytokines for 12 h. Cells were then collected for the extraction of protein. The expression of phosphorylated STAT3 (p-STAT3), total STAT3, phosphorylated p65 (p-p65) and GAPDH were analyzed by western blot. A representative of three experiments is shown. **b** C57BL/6 MSCs were treated with indicated cytokines and molecules for 12 h. Cells were then collected and the expression of p-STAT3 and total STAT3 were detected by western blot. **c** C57BL/6 MSCs were treated by indicated cytokines with or without WP1066 (5 μM). After 12 h, cells were collected for the measurement of STAT3 phosphorylation by western blot. **d** C57BL/6 MSCs were treated by indicated cytokines with or without WP1066 for 24 h. Cells were then collected for the detection of PD-L1 mRNA by real-time PCR

To elucidate the molecular mechanism by which IL-17 promote PD-L1 expression on MSCs, we test the effect of IL-17 on transcription factors responsible for PD-L1 expression. NF-kB and STAT3 are best characterized transcriptional factors which were reported to regulate PD-L1 [41–43]. We thus examined the activity of NF-kB and STAT3 upon IL-17 stimulation. We found that although p65 phosphorylation was increased upon the stimulation of IFNγ and TNFα, the addition of IL-17 did not further strengthen this signal. On the other hand, IL-17 dramatically enhanced the phosphorylation of STAT3, indicating NO may act through STAT3 to promote PD-L1 expression (Fig. 4a). To further validate this result, we examined whether NO donor also regulate PD-L1 in a STAT3 dependent manner. We found in the presence of IFNγ and TNFα, SNAP enhanced the signal transduction by STAT3 (Fig. 4b). Interestingly, SMT could also induce STAT3 phosphorylation while failed to boost PD-L1 expression. A possible explanation which reconcile the contradictory observations is that p-STAT3 signaling is indispensable but not sufficient to induce PD-L1 expression. NO may also corporate with p-STAT3 signaling to boost PD-L1 expression through other signaling pathways in MSCs. Further investigations are required to fully address this important issue.

SNAP alone can also activate STAT3, however, it cannot induce PD-L1. To prove the cause effect between enhanced STAT3 signaling and upregulated PD-L1 expression, we employed STAT3 inhibitor WP1066 which can efficiently suppress the activity of STAT3 pathway (Fig. 4c). We found that when the STAT3 phosphorylation was inhibited, the expression of PD-L1 was also dramatically depressed (Fig. 4d). These experiments showed that IL-17 promoted PD-L1 expression through NO-activated STAT3 pathway.

### IL-17 pretreated MSCs promote tumor growth through PD-L1

**Fig. 5:**
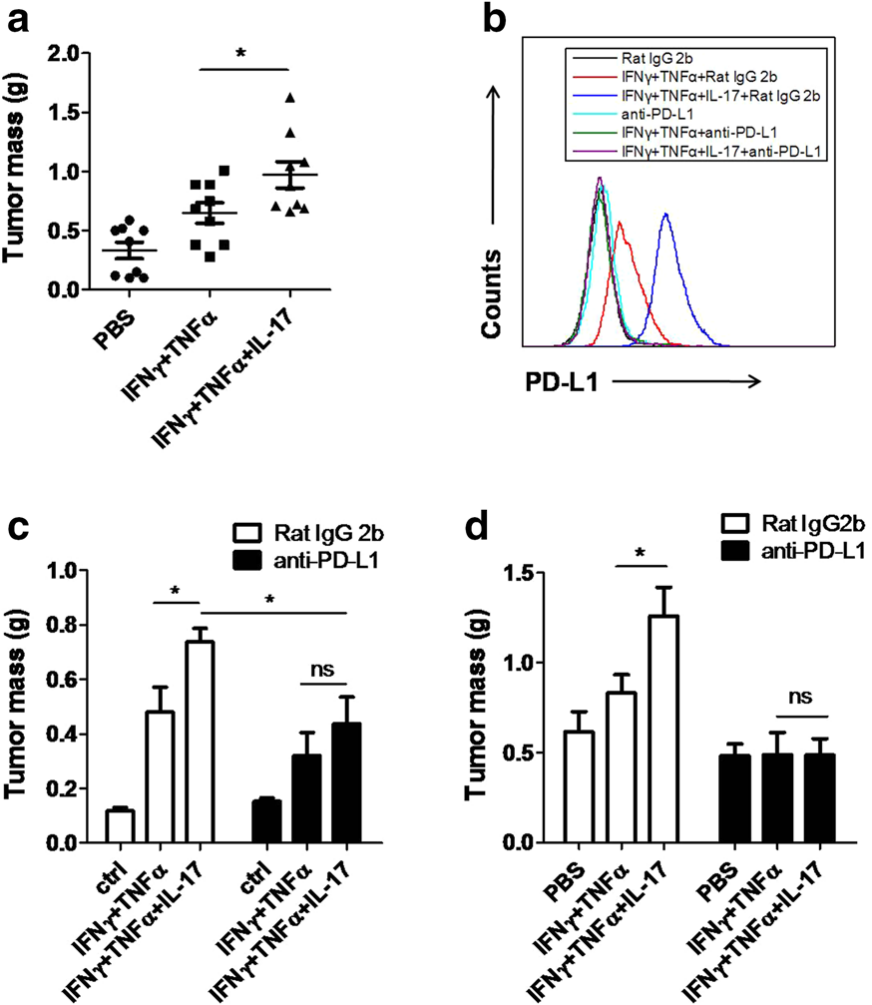
IL-17-pretreated MSCs promote melanoma growth through enhancing PD-L1 expression on MSCs. **a** C57BL/6 MSCs were treated by PBS, or IFNγ and TNFα, or IFNγ, TNFα and IL-17 for 48 h. Pretreated MSCs (1 × 10^5^) were then collected and injected subcutaneously together with B16F0 cells (2.5 × 10^5^) on C57BL/6 mice (n = 9 each). On day 16, mice were euthanized and the resultant tumors were excised and weighed. **b** C57BL/6 MSCs were treated as in **a** with or without PD-L1 antibody (10 μg/ml). After 48 h, cells were collected for the detection of PD-L1 by flow cytometry. **c** C57BL/6 MSCs collected in **b** were then injected with B16 cells as in **a** (n = 5 each). On day 16, mice were euthanized and the resultant tumors were excised and weighed. **d** C57BL/6 MSCs (1 × 10^5^) and B16 cells (2.5 × 10^5^) were injected as in **a**. On day 1,4 and 7, PD-L1 antibodies (10 μg/g) were administrated intraperitoneously (n = 6 each). On day 16, mice were euthanized and the resultant tumors were excised and weighed. Statistical analysis was performed using the Student’s t-test. *p < 0.05, ns (not significant)

Our in vitro data showed IL-17 could dramatically enhanced PD-L1 expression on MSCs. Considering the importance of PD-L1 in tumor progression and the presence of MSCs in the tumor microenvironment, it is possible that IL-17 could promote tumor growth through upregulating PD-L1 expression on MSCs. To test whether IL-17 shapes the tumor promoting effects of MSCs, we established melanoma model through the administration of B16F0 tumor cells together with cytokine-pretreated MSCs, and then compared the effect of MSCs with or without IL-17 on tumor growth. We found that the administration of MSCs with IL-17 pretreatment significantly promoted melanoma growth (Fig. 5a). To verify whether the tumor promoting effects of IL-17 primed MSCs are dependent on PD-L1, we employed PD-L1 neutralizing antibody to block ligation site of PD-L1 (Fig. 5b). In this condition, we found that IL-17 primed MSCs can no longer promote tumor growth in vivo (Fig. 5c). To further confirm this result, we also administrated PD-L1 antibodies to tumor bearing mice. As expected, the effect of IL-17 pretreated MSCs on tumor growth is completely inhibited (Fig. 5d). Collectively, our data suggested that IL-17 can act through MSCs to promote melanoma growth.

## Discussion

In the last decade, it has been established that MSCs participate in tumor development and metastasis through shaping tumor microenvironment [14]. Substantial evidence showed that MSCs express a plenary of molecules to exert their tumor promoting effects, either by suppressing cytotoxic immune responses or promoting the recruitment of immunosuppressive macrophages [22]. We found that IL-17 can synergize with IFNγ and TNFα to promote PD-L1 expression of MSCs. IFNγ, TNFα and IL17 commonly function as pro-inflammatory cytokines. IFNγ and TNFα are mainly secreted by immune cells, such as lymphocytes and NK cells, and act as an important inducer of various cytokine and chemokine genes [44, 45]. Ironically, these pro-inflammatory cytokines are also present in immune paralysis tumor microenvironment. Our previous studies showed that TNFα treated MSCs can express chemokines to recruit M2-like macrophages to promote tumor growth, suggesting these generally pro-inflammatory cytokines could induce immunosuppression through MSCs. In the current study, we showed this is also true with IL-17. Therefore, it is important to interpret the biological effects of cytokines in the context of MSCs.

In our study, we have shown that NO is responsible for the enhanced PD-L1 expression on MSCs, since genetic deletion of iNOS gene and iNOS inhibitor could suppress the PD-L1 expression. Notably, the suppression of NO production did not completely inhibit the expression of PD-L1 as we expected, but down to the same level of that as treated with IFNγ and TNFα. This indicates that PD-L1 gene expression in MSCs is induced by IFNγ and TNFα and further enhanced by NO accumulation. Without the signal of IFNγ and TNFα, NO alone could not induce PD-L1 expression. This conclusion is further verified by the fact that NO donor alone showed no effect on PD-L1 expression, while together with IFNγ and TNFα, NO can act as the substitute of IL-17 to enhance the expression of PD-L1.

NO is reported to regulate the expression of various cytokines, growth factors and chemokines, including IL-6, IL-10, IFNγ, TGFβ, M-CSF, VEGF and MCP-1, which are important in tumor growth and metastasis [46]. Here, we reported that NO induced expression of PD-L1 on MSCs through activating STAT3. However, detail studies are still required to fully illustrated how NO promote STAT3 phosphorylation. The effect of NO on protein is often achieved through introducing a nitro group (–NO_2_) to amino acids, mainly tyrosines [47]. This tyrosine nitration process will result in directly gain or inhibition of protein function, or indirectly influence the phosphorylation process to further regulate protein function. Therefore, it is possible that in MSCs, NO can result in the nitration of STAT3, which in turn increases the phosphorylation of STAT3. This study would also provide novel information of NO-mediated inhibition of immune responses.

Our studies showed that IL-17 pre-treated MSCs can promote tumor growth through enhanced PD-L1 expression. PD-L1 exerts its effect mainly through directly inhibiting lymphocyte activation and proliferation, especially CD8+ T cells. It is worth investigating that how these PD-L1 expressing MSCs regulates tumor microenvironment. It is reported that MSCs often promote tumor progression through Treg induction. An important function of PD-L1 is to upregulate Treg. Therefore, the Treg induced by tumor-associated MSCs may due to its PD-L1 expression. This merits further investigation.

In conclusion, the results presented here suggest that IL-17 can synergize with IFNγ and TNFα to promote PD-L1 expression on MSCs. Enhanced expression of PD-L1 on MSCs within tumor microenvironment will result in increased tumor growth. Thus, we have proposed a new mechanism in which IL-17 act through MSCs to promote tumor growth, which enriched our understanding of the relationship between IL-17 and tumor growth and due to the important role of PD-L1 in controlling immune responses, our study also provided novel information for the application of IL-17 treated MSCs in not only cancer, but also inflammatory diseases.

## Methods

### Animals

C57BL/6 mice were purchased from the Shanghai Laboratory Animal Center of Chinese Academy of Sciences, Shanghai, China, and maintained under specific pathogen-free (SPF) conditions. iNOS^−/−^ mice were obtained from Jackson Laboratory (Bar Harbor, ME, USA). Mice were housed in the Vivarium of Shanghai Jiao Tong University School of Medicine. All procedures were approved by the Institutional Animal Care and Use Committee of the Institute of Health Sciences, Shanghai Institutes for Biological Sciences of Chinese Academy of Sciences. Animals were matched for age and gender in each experiment.

### Antibodies and reagents

*S*-nitroso-*N*-acetyl-penicillamine (SNAP), PMSF and Griess reagent were from Sigma-Aldrich (St. Louis, MO, USA). Recombinant mouse IFNγ, TNFα, IL-17A and anti-mouse CD274 (PD-L1)-APC were from eBiosciences (La Jolla, CA, USA). WP1066 was from Santa Cruz Biotechnology (Dallas, TX, USA). Antibodies against GAPDH, p-p65, p-STAT3 and STAT3 were from Cell Signaling Technology (Danvers, MA, USA). S-methyl-isothiourea (SMT) was from Beyotime Technoloby (Shanghai, China).

### Cells

MSCs were generated using our previously described protocol. Briefly, tibia and femur bone marrow of 6-week-old wild-type or iNOS^−/−^ mice was harvested. Cells were cultured in DMEM medium supplemented with 10% FBS, 2 mM glutamine, 100 U/ml penicillin, and 100 μg/ml streptomycin (complete medium, all from Invitrogen, Carlsbad, CA, USA). All non-adherent cells were removed after 24 h (hr), and adherent cells were maintained. Medium was changed every 3 days. To obtain MSC clones, cells at confluence were harvested and seeded into 96-well plates by limited dilution. Individual clones were then picked and expanded. These MSCs were capable of differentiating into adipocytes and osteocytes under the respective differentiation conditions. Cells were used before the 15th passage. B16F0 mouse melanoma cells were cultured in DMEM medium supplemented with 10% FBS, 2 mM glutamine, 100 U/ml penicillin, and 100 mg/ml streptomycin.

### Flow cytometric analysis

MSCs were treated with or without different combinations of IFNγ (2 ng/ml), TNFα (2 ng/ml), IL-17A (10 ng/ml), SMT (1 mM) and SNAP (1 mM). 2 days later, cells were digested and harvested and then suspended at a concentration of 2 × 10^6^ cells/ml (in PBS with 2% FBS) and stained by anti-mouse CD274 (PD-L1) APC antibodies for 30 min. Antibody-stained cells were washed twice with PBS. Fluorescence intensity was measured by flow cytometry (FACS Calibur, BD Immunocytometry).

### Real-time PCR

Total RNA was isolated using RNAprep pure Cell/Bacteria Kit (Tiangen biotech, Beijing, China). First-strand cDNA synthesis was performed using PrimeScript™ RT Master Mix (TaKaRa Biotech, Dalian, China). The levels of mRNA of genes of interest were measured by real-time PCR (7900 HT by Applied Biosystems, Foster City, CA, USA) using SYBR Green Master Mix (TaKaRa Biotech, Dalian, China). Total amount of mRNA was normalized to endogenous β-actin mRNA. Sequences of PCR primer pairs were as follows: mouse PD-L1, forward 5′-GCTCCAAAGGACTTGTACGTG-3′ and reverse 5′-TGATCTGAAGGGCAGCATTTC-3′; mouse iNOS, forward 5′-CAGCTGGGCTGTACAAACCTT-3′ and reverse 5′-CATTGGAAGTGAAGCGTTTCG-3′; mouse β-actin, forward 5′-CCACGAGCGGTTCCGATG-3′ and reverse 5′-GCCACAGGATTCCATACCCA-3′.

### Detection of NO

NO was detected using a modified Griess reagent. Briefly, 50 μl supernatant was mixed with equal volumes of Griess reagent, and the concentration of total NO_2_ in the supernatant was detected by the Griess reaction through reading the absorbance at 540 nm after 15 min.

### Western blotting

Cells were washed twice with ice-cold PBS, harvested and lysed in the RIPA buffer (Millipore, Temecular, CA, USA) containing a cocktail of protease inhibitors (Roche, Natley, NJ, USA) and PMSF for 30 min on ice. Lysates were clarified by centrifugation at 16,000×*g* for 15 min and heated in sodium dodecyl sulfate sample buffer at 95 °C for 10 min. Protein concentration of the supernatant was determined by the Bradford assay (Bio-Rad, Hercules, CA, USA). Protein samples were separated on a polyacrylamide gel, and separated proteins were electroblotted onto polyvinylidene difluoride membranes. Specific proteins were revealed by mouse and rabbit antibodies against p-STAT3, STAT3, p-p65 or GAPDH by overnight incubation at 4 °C, followed by chemiluminescent detection according to the manufacturer’s instructions.

### Mouse tumor model

MSCs were pretreated with IFNγ and TNFα, or IFNγ, TNFα and IL-17A with or without 10 μg/ml PD-L1 antibodies. 2 days later, cells were digested, washed and harvested for tumor model. Each mouse was injected with 2.5 × 10^5^ B16F0 in 100 μl PBS subcutaneously with or without pretreated MSCs (1 × 10^5^). Mice were observed daily. On day 16 after tumor cell administration, the resultant tumors were then excised and weighed. Each experimental group included at least five mice.

### Statistical analysis

Data are presented as mean ± SEM. Statistical significance was assessed using unpaired two-tailed Student’s t-test, *p < 0.05, **p < 0.01, or Log-Rank test in survival experiment: **p < 0.01, ***p < 0.001.

## Data Availability

Please contact the corresponding author for data on reasonable request.

## Abbreviations

IFNγ: Interferon-γ
IL-17: Interleukin-17
iNOS: Inducible nitric oxide synthase
MSCs: Mesenchymal stem cells
NO: Nitric oxide
PD-L1: Programmed death-ligand 1
SMT: *S*-methyl-isothiourea
SNAP: *S*-nitroso-*N*-acetyl-penicillamine
STAT3: Signal transducer and activator of transcription 3
TNFα: Tumor necrosis factor-α
